# Don’t add pressure to stress: Measured water potentials differ by method under severe drought

**DOI:** 10.1093/plphys/kiaf634

**Published:** 2025-12-07

**Authors:** Laura Fernández-de-Uña

**Affiliations:** Assistant Features Editor, Plant Physiology, American Society of Plant Biologists, Rockville, MD, United States; Department of Plant Biology and Soil Sciences, Universidade de Vigo, Ourense, Spain

Water potential (Ψ) measures water energy and results from the combination of different drivers of water movement, including turgor pressure, osmotic water potential (related to solute concentrations), gravity, and matric water potential (related to how water binds to surfaces—eg, capillarity). Water potentials are the driving force of water transport along the soil-plant-atmosphere continuum and a key measure of the plant's water balance. According to the cohesion-tension theory, water ascends under tension from the roots to the leaves along xylem conduits thanks to the negative hydrostatic pressure generated at the leaf surface as a result of water evaporation (Hacke and Sperry 2001). Therefore, plant Ψ are measured in pressure units (generally MPa) and take negative values in such way that higher absolute values (ie, more negative) indicate that xylem conduits are withstanding greater pressures.

Plant Ψ are largely driven by soil Ψ (directly linked to water availability), becoming more negative as drought progresses. Increasing water deficits can induce xylem cavitation if air bubbles are aspirated into water-filled conduits, thus producing an embolized, nonfunctional conduit. Extensive cavitation disrupts sap supply to the leaves, causing desiccation and potentially plant death (Hacke and Sperry 2001; Choat et al. 2012). Plants thus adjust xylem Ψ through stomatal closure, preventing extensive water loss and stress-induced embolism. However, as drought progresses, stomatal closure can no longer prevent the drop in xylem Ψ, and the plant may suffer hydraulic failure (Choat et al. 2012).

Species operate within different hydraulic safety margins (ie, how close minimum xylem Ψ get to water potentials causing severe water transport disruption) (Choat et al. 2012). Therefore, measurements of xylem Ψ can be used to establish critical points in plant function as water deficits advance. Xylem hydraulic vulnerability curves establish a sigmoidal relationship between xylem Ψ and the associated loss in xylem conductivity (a measure of water transport capacity) as a result of conduit embolism. Critical points are extracted from this curve, namely the water potentials at which air stars to enter the xylem (P_e_) and at which 50% (P50) and 88% (P88) of the conductivity is lost, marking a significant risk of drought-driven hydraulic failure (Domec and Gartner 2001; Choat et al. 2012). Similarly, pressure-volume curves provide information on the water potentials at which leaves start wilting (turgor loss point) (Bartlett et al. 2012). Therefore, accurate xylem Ψ measurements provide key information on plant performance under drought and the triggers of drought-induced mortality.

Plant tissue Ψ can be directly measured using thermocouple psychrometers. These instruments measure the vapor pressure within a sealed chamber in equilibrium with the sample, equivalent to its water potential (Turner 1981). However, due to the cost of these sensors for in situ, continuous measurements, Ψ are generally measured indirectly using a pressure chamber (Scholander et al. 1965). In this method, an excised, nontranspiring leaf or small-diameter shoot is enclosed in a chamber, with its petiole out and its bark and phloem removed, and pressure is applied until xylem sap is extracted (Fig. A). That pressure is assumed to be the sample's water potential. Two key assumptions are made: (1) if the leaf is not transpiring (eg, in the dark), there is an equilibrium between leaf (Ψ_leaf_) and stem (Ψ_stem_) water potential, and therefore Ψ_stem_ equals Ψ_leaf_; and (2) the osmotic, gravitational, and matric components of Ψ are considered negligible so that the pressure obtained represents the tension component of xylem Ψ (Turner 1981). However, it has been found that water potentials causing leaf xylem embolism may also result in leaf tissue damage (Brodribb et al. 2021), and thus leaf pressurization may extract the content of damaged cells, altering leaf osmotic potential and in turn invalidating the second assumption of pressure chamber measurements.

**Figure 1.**
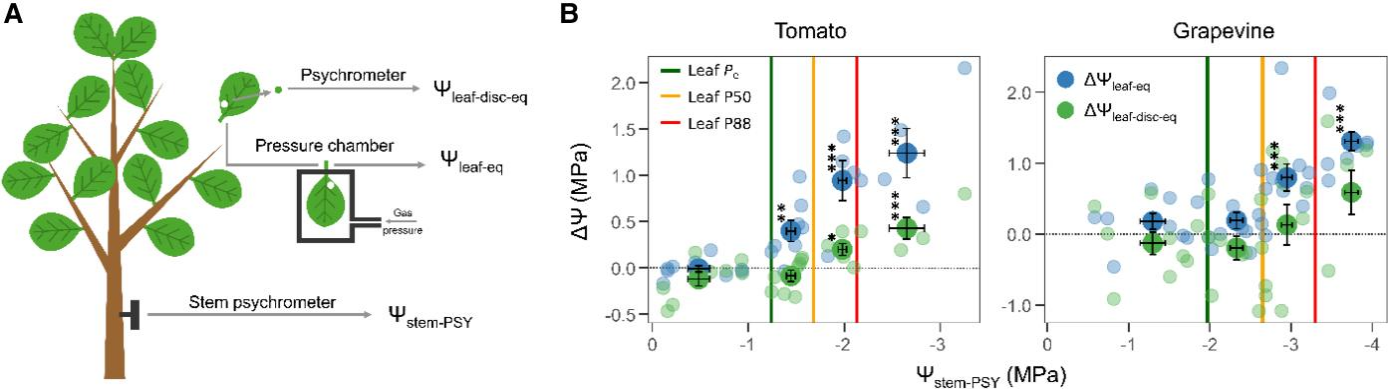
**A)** Different measurements taken by Rodriguez-Dominguez et al.: stem water potentials measured with a psychrometer (Ψ_stem-PSY_), leaf water potentials measured in leaf discs with a psychrometer (Ψ_leaf-disc-eq_), and leaf water potentials measured with a pressure chamber (Ψ_leaf-eq_). **B)** Differences between Ψ_stem-PSY_ and Ψ_leaf-eq_ (ΔΨ_leaf-eq_; blue points) and Ψ_stem-PSY_ and Ψ_leaf-disc-eq_ (ΔΨ_leaf-disc-eq_; green points) along the gradient in Ψ_stem-PSY_ shown in Rodriguez-Dominguez et al. Fainted points represent individual measurements and bigger, darker points represent the average within 4 water potential ranges delimited by the stem water potentials at which air stars to enter the xylem (P_e_; green vertical line), 50% of the conductivity is lost (P50; yellow line) and 88% of the conductivity is lost (P88; red line). The dashed horizontal line marks a difference of 0 between measurement types (ie, both methods give the same result). Asterisks indicate statistically significant differences with *P* < 0.05 (*), *P* < 0.01 (**), and *P* < 0.001 (***) (figure adapted from Rodriguez-Dominguez et al. 2025).

Recently in *Plant Physiology*, Rodriguez-Dominguez et al. (2025) compared stem water potentials measured directly with a psychrometer (Ψ_stem-PSY_) with leaf water potentials measured directly in leaf discs using a psychrometer (Ψ_leaf_-_disc-eq_) and indirectly with a pressure chamber (Ψ_leaf-eq_; Fig. A) in 2 species with contrasting responses to drought—tomato (*Solanum lycopersicum*), a herbaceous species, and grapevine (*Vitis vinifera*), a woody species—to establish the range of Ψ_stem_ that can be accurately measured using indirect methods in individuals subjected to dehydration.

For both species, Ψ_stem-PSY_ was highly correlated to both measurements of leaf water potential, but there was not an exact correspondence between Ψ_stem-PSY_ and Ψ_leaf_ measurements, with Ψ_leaf-disc-eq_ being closer than Ψ_leaf-eq_ to Ψ_stem-PSY_ (Rodriguez-Dominguez et al. 2025). When the authors assessed the difference between Ψ_stem-PSY_ and Ψ_leaf-eq_ (ΔΨ_leaf-eq_) and Ψ_stem-PSY_ and Ψ_leaf-disc-eq_ (ΔΨ_leaf-disc-eq_) along the gradient in Ψ_stem-PSY_, they found that these differences were close to 0 under moderate Ψ_stem-PSY_ but increased (ie, Ψ_stem-PSY_ was more negative than Ψ_leaf_) after certain leaf embolism thresholds were surpassed (Fig. B). A lower amount of leaf xylem embolism was needed to dissociate pressure chamber measurements (Ψ_leaf-eq_) from Ψ_stem-PSY_ (starting as low as P_e_ for tomato and P50 for grapevine) than psychrometer measurements (Ψ_leaf-disc-eq_; P50 for tomato and P88 in grapevine, although in the latter the difference was not statistically significant from 0). The higher values of Ψ_leaf-eq_ than Ψ_stem-PSY_ indicate that sap exited the petiole under pressure at less negative water potentials than it should, likely because pressurizing leaves drew out the contents of cells that had been damaged by cavitation (Brodribb et al. 2021). This hypothesis was further supported by the observed decline in the osmotic water potential of the leaf xylem sap extracted during pressurization in both species, suggesting a greater presence of solutes possibly coming from disrupted cells (Rodriguez-Dominguez et al. 2025).

Rodriguez-Dominguez and colleagues demonstrate that indirect measurements of Ψ_stem_ are sufficiently accurate at moderate water potentials, but direct and indirect measurements may dissociate when Ψ approach the point of leaf damage. These results indicate that pressure chamber Ψ_leaf_ measurements may overestimate the Ψ_stem_ levels leading to xylem disruption, at least in the study species. Given that pressure chamber Ψ_leaf_ measurements are commonly used to construct xylem vulnerability and pressure-volume curves and to subsequently establish important thresholds such as P50, P88, and turgor loss point, caution should be taken when determining very negative water potentials close to those inducing leaf tissue damage, avoiding the use of leaves with damaged tissue. Nonetheless, the observed methodological discrepancies are not universal (eg, Gauthey et al. 2020), and therefore further studies are needed to establish what species or functional types are susceptible to biased Ψ_leaf_.

## Data Availability

No new data were generated or analyzed in support of this article.

## Recent research articles in *Plant Physiology*

- Carins-Murphy et al. (2023) found that high floral transpiration induced by heat and water stress led to xylem failure and tissue damage in pyrethrum flowers (https://doi.org/10.1093/plphys/kiad349).
- Bristow & Knipfer (2025) provided an extension of the triphasic water potential curve by adjusting ΨMD for vapor pressure deficit to predict the threshold of complete stomatal closure and TLP associated with soil water deficit (https://doi.org/10.1093/plphys/kiaf337).
